# Travel Time for Dental Care Services Based on Patient Preference in South Korea

**DOI:** 10.3390/ijerph19042051

**Published:** 2022-02-12

**Authors:** Han-A Cho, Bo-Ra Kim, Hosung Shin

**Affiliations:** 1 Department of Dental Hygiene, Shinhan University, 95, Hoam-ro, Uijeongbu-si 11644, Korea; choruchia@naver.com; 2 Department of Dental Hygiene, Namseoul University, 91, Cheonan-si 31020, Korea; violetbo@naver.com; 3 Department of Social and Humanity in Dentistry, Wonkwang University School of Dentistry, 460, Iksan-daero, Iksan-si 54538, Korea

**Keywords:** dental care, dental health services, patient preference, travel time

## Abstract

This study analyzed patient preferences using travel time from residence to dental institution when selecting dental care services. We used data from the Korean Health Panel from 2008 to 2017 and analyzed each dental service episode. Since the distribution of travel time was skewed to the left, median travel time was analyzed. The association of travel time with services was analyzed via the population-averaged generalized estimating equation (GEE) with the Poisson family. The median of the average travel time per episode was longer for non-National Health Insurance (NHI)-covered services and shorter for NHI-covered services. The first quintile of low-income subjects traveled the longest for all services and utilized dental care the most. In the GEE analysis, travel time was approximately three times longer for implant treatment and gold inlay/resin fillings and >2 times longer for orthodontic care than for NHI-covered services. Patients residing in rural counties traveled for longer than residents of large cities. Income was statistically significant; however, the coefficient was close to zero. Travel time was related to the type of service and reflected patient preference. This was more prominent for expensive non-NHI-covered services than for NHI-covered services. The findings suggest patients’ subjective preferences for dental clinic selection are expressed as rational deliberation considering each individual’s situation.

## 1. Introduction

Issues in healthcare access include travel time, convenience, choice of provider, quality, and cost of care [1,2]. These factors were related to the evolution from potential access to realized access and focused on the interaction between key elements that determined the use of healthcare services [3]. Penchansky described access to health care in terms of five dimensions: availability, affordability, accommodation, acceptability, and accessibility [4]. Availability is related to the adequacy of the supply. Affordability is related to a patient’s ability to pay for healthcare services. Accommodation describes the organization and content of the healthcare system as it relates to clinical hours, waiting time, and transportation. Acceptability represents the attitudes toward providers, which are related to provider–patient relationship, number of visits for treatment, quality of care, etc. Accessibility, which is related to the geographic location of a healthcare institution, is often measured in terms of travel time, distance, travel cost, and the effort required to reach the facility [4,5].

In general, patients are reluctant to increase travel time for the treatment they want [6,7], and they prefer to seek care from a provider near their residence [6]. Research indicates that patients more often tend to visit providers located in close proximity to their homes [8]. Conversely, when patients want to receive treatment from a high-quality provider [7,9] or a healthcare institution with a good reputation [10], they have been shown to be willing to endure the additional travel time [9] and opportunity cost burden. For non-emergency and elective treatments, the expertise of the healthcare provider (or a need for specialized care) also has an effect on patient travel time. For example, low-income children and adolescents have been shown to travel longer distances to receive orthodontic treatment than others [11]. Users of dental treatment for non-covered services of the National Health Insurance (NHI; hereafter ‘non-NHI-covered’) in South Korea, which includes mostly non-emergency and elective treatments (e.g., implants/orthodontics and dental prostheses) traveled further to receive treatment, bypassing the dental office nearest their residence [12,13].

In terms of the bypass concept, many studies have analyzed the preferences of patients using healthcare services in rural and remote areas [14,15,16], at residences and workplaces [8], in limited groups [11,17], and in a dental setting [12]. Patient preference in choosing a healthcare institution is based on individual perception and reflects the patient’s healthcare wishes. Both preference and decision are reported to be related to health literacy, numeracy, and locus of control [18]. Therefore, patient preference involves the relative desirability or acceptability of healthcare services or providers, resulting in the patient entertaining tradeoffs between the factors considered when making individual decisions [5]. To actively select providers that best suit their preferences and needs, patients typically compare the price and quality of healthcare providers [5,19]. Studies also suggest that the choice of healthcare provider can be an expression of patient expectations of how a health facility improves on its service delivery, and the satisfaction of the patient’s own prior experience, or that of others [5]. However, because expectations when selecting a healthcare institution can affect patient satisfaction, continuous and effective management of patient expectations is required to reduce negative evaluations of treatment experience [20].

Anderson argued that realized access was related to type, site, purpose, and coordinated services received [3]. Travel time in this study is a measure of spatial proximity to the dental institution for realized access. Patient preferences for certain providers are expressed as choosing a specific healthcare institution among numerous alternatives, taking into account all the factors involved in that choice, such as Penchansky’s five terms for access to care. From the patient’s viewpoint, these terms include the spatial proximity to a specific healthcare institution (i.e., travel time). In this study, travel time reflects the patient’s preference for obtaining dental treatment. The travel time to reach a dental clinic to receive the dental care service was analyzed to identify the difference between dental service types, such as non-NHI-covered services and NHI-covered services.

### Dental Healthcare in South Korea

As of 2018, the average DMFT (Decayed, Missing, and Filled Teeth) experienced by 12 year-old children was 1.84, which is higher than the average of 1.2 in OECD member countries [21]. National Health Insurance (NHI) is a healthcare insurance system operated by a single insurer that is compulsory for all South Korean citizens (living in S. Korea). In terms of the dental benefits, most conservative treatments, periodontal treatments, and oral surgeries are covered, but orthodontic, prosthetic, and preventive services are not covered. As a result, patients paid out-of-pocket costs corresponding to 63–73% of the total dental expenses, although the co-pay for NHI-covered benefit items is 30% [22,23,24]. Since 2012, dental benefits have been expanded in stages, providing preventive services for children, as well as complete and partial dentures once per lifetime and up to two implants for the elderly [25]. Of the elderly population aged 65 years and older, 41.6% received NHI-covered dentures or dental implants. Among them, implants accounted for 23.7% of the elderly population [26]. Recently (2021), orthodontic treatment was provided only to patients with congenital orofacial anomalies. The co-pay for orthodontic treatment is 30% by default, but there is a slight difference depending on the socioeconomic status of the patient [27]. 

In 2019, the number of dentists per 1000 of the population was 0.5, which is lower than the OECD average [28]. However, the annual number of outpatient dental visits per capita was 1.4, which is slightly higher than the OECD average [29]. According to existing research, most studies revealed that dentists in South Korea were in a state of oversupply [30,31]. However, they were concentrated in highly populated areas, such as large cities; in rural areas, patients traveled an average of twice as long to visit dental facilities [12]. Since the dental specialist system began in 2011, 11 dental specializations have been established. In 2018, the number of dental specialists increased sharply, and 30% of all active dentists were specialists. Among them, 17.6% were prosthetists and 17.7% were orthodontists [29]. However, currently, the referral system between primary and secondary dental care is not clearly divided in the dental care delivery system. As a result, more than 90% of dentists perform the role of primary care dentist [24]. There is no distinction between private dentistry and university-based dentistry, and patients choose university dental hospitals or private clinics according to their preference. However, there is a difference in the cost of treatment for the same service. At the municipal level, public health centers are installed in South Korea. These public institutions initially aimed to provide healthcare services for people living in disadvantaged areas. However, in recent years, rather than providing healthcare services, these institutions have provided local public health services [32]. 

Choosing a dental facility is a free choice. The total cost, including travel expense, is borne by the individual. Private dental insurance, which is supplemental insurance and has about 1 insured person for every 10 aged 20 to 50, offers various dental plans depending on the treatment details, dental materials, and the age of the insured individual, but transportation expenses are not included in the benefit details [33].

## 2. Materials and Methods

This study was approved by the Institutional Review Board (WKIRB-202102-SB-006) of Wonkwang University.

### 2.1. Data and Subjects

This study is a longitudinal study, and data from the Korea Health Panel (KHP) (version 1.6) were used for the 10 years from 2008 to 2017, inclusive. The KHP is a representative health care panel survey that can analyze in-depth not only information on healthcare use behavior and expenditure but also factors affecting healthcare use and expenditure. The KHP periodically conducts the same questionnaire every year for the same households across the country. In order to collect data for KHP, a surveyor in charge of panel households is assigned first (one surveyor per about one-hundred households), and education is provided on the contents of the KHP survey and the questionnaire guideline. Next, the investigator makes a household visit and performs a CAPI (Computer-Assisted Personal Interview)-based survey. When surveyors visit target households, they collect healthcare expense or prescription drug receipts related to the household’s healthcare with participants’ health account books to prevent omissions or errors. The KHP collects personal and household data on the socioeconomic characteristics of households and household members, income, living expenses, and private healthcare insurance subscription details, as well as detailed descriptions of annual total healthcare use and expenditure, including dental services. It also has the advantage of tracking changes in healthcare use and expenses according to disease and type of service [34].

Starting with 7866 households and 24,616 household members in 2008, a sample of approximately 82% was active, including 17,184 household members of 6408 households in 2017. This study used 53,963 dental episodes of 7681 households, 16,493 household members, and 173,863 cases for 10 years of dental treatment services. The unit of analysis in this study was defined following the protocol of Korea’s Standards of Medical Care Benefits (KSMCB) [35]. According to KSMCB, the last visit within 90 days due to relevant services was classified as the same episode, but if the visit for the same disease exceeded 90 days, it was considered as a new episode. Based on those judgments, all dental treatments were recalculated in units of episodes.

### 2.2. Variables

Sex, age, marital status, educational attainment level, economic activity, household income, private dental insurance, medical aid, regionality, and dental service type were selected as independent variables. Age was divided into three categories: children, <20 years of age; adults, 20 to ≤65 years; and elderly, >65 years. Marital status was categorized as married or single [22]. Educational attainment level was classified into three categories: middle school graduation or lower, high school graduation, and college or higher [13,22]. For income, a square root equivalence scale that considered the household’s total earned income, total asset income, and the number of household members for one year was used [36]. In the case of economic activities, the existence of a job was checked regardless of regular or non-regular status [22,37]. Private dental health insurance and medical aid were dichotomized [37,38]. To check the distribution of dental resources by geographical region, the participant’s living areas were divided into large city districts, small- and medium-sized cities, and rural counties [13].

The dental services under the NHI-covered dental benefits in this study include: (1) amalgam filling and composite resin filling; (2) periodontal treatment after diagnosis of gingivitis and periodontal disease; (3) endodontic treatment; (4) all extraction procedures, including alveoloplasty; (5) sealant treatment for individuals under the age of 18 as a preventive treatment; and (6) scaling for preventive purposes [13,39]. When classifying the type of dental treatment, the study used the KHP questionnaire. The dental treatment variables were classified as follows: (1) NHI-covered dental service, (2) denture and fixed bridge, (3) gold inlay and resin filling, (4) implant, (5) orthodontic, (6) teeth whitening, and (7) others (e.g., oral health education, oral examination, and temporomandibular treatment). NHI-covered services were aggregated into one because out-of-pocket expenses were low and would not act as a factor in terms of travel burden for seeking a preferred dental clinic. Patients in Korea preferred to visit dental clinics near their residences for NHI-covered dental services. The travel mode was divided into by walking and by vehicle (using a private vehicle, taxi, public transportation, train/airplane, and free shuttle bus). 

The dependent variable, travel time [40], was calculated using the patient’s average visit time per dental episode and reflected the following question: “How many minutes did it take to get from home (or work) to the dental office (hospital)?” In this case, ‘dental episode’ refers to the episode organized according to the definition given above. Studies examining healthcare utilization behavior often use geographical information systems, which can underestimate the actual travel distance or travel time due to traffic congestion or traffic signal systems [41,42]. The authors concluded that the patient’s direct response on the questionnaire expressed more accurate spatial proximities to dental institutions.

### 2.3. Analyses

The median test was performed to confirm the characteristics of the participants due to the left-skewed distribution. The population-averaged generalized estimating equation (GEE) was used to estimate the association between dental services and travel time. In this study, the choice of dental clinic was related to the particular dental service that a patient would like to receive. That is, patients tended to choose dental clinics carefully when obtaining expensive treatments, such as non-NHI-covered services, while NHI-covered services were not chosen as carefully in comparison. For repeated measurements, GEE is often used to consider intra-individual correlation [43,44,45]. The GEE in this study estimates the population-based average influence of travel time on dental service selection. For privacy protection reasons, the participants’ geographical region variables contained some missing values (about 15% of episodes); thus, the analysis was conducted without these missing values. All analyses were performed using the R software (R foundation for Statistical Computing, Vienna, Austria) package (R version 4.0.0, www.r-project.org (accessed on 28 April 2021)).

## 3. Results

### 3.1. General Participant Characteristics

NHI-covered dental services were the most common, followed by denture and fixed bridge, others, and orthodontics. Female, 20–65 year-old, single, middle/high-school-educated level, and employed participants sought more dental services than those of the reference. Those who subscribed to private dental insurance and had medical aid sought a small number of dental episodes because the number of people in the group was small.

The median of the average travel time per episode of gold inlay and resin filling was the longest, regardless of travel mode, followed by implants. Conversely, the median of the average travel time per episode of NHI-covered treatment was the shortest. Older age, single, unemployed, medical aid recipients and low-income groups (first quantile of equivalent income, Q1) traveled for a relatively long time. In geographical regions, the median average travel time per episode in the rural counties was the longest. Private dental insurance did not show a statistically significant meaning (Table 1). 

### 3.2. The Average Visit Number Per Episode by Income Quintile

**Figure 1:**
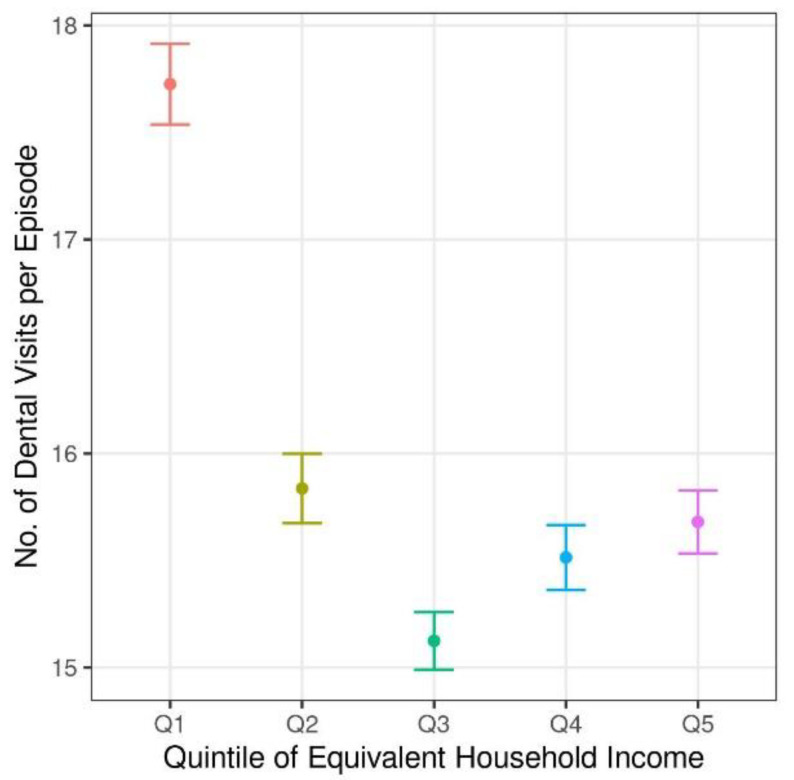
The average frequency of total dental episode by income quintile.

The average frequency of dental visits per episode in the income quintile for 2008–2017 was analyzed (Figure 1). The first income quintile (Q1) refers to the lowest income group, and the fifth quintile (Q5) is the highest. The average frequency of dental visits per episode was highest in Q1, and the third quartile was the lowest. Here, the average frequency of dental visits per episode in Q5 was higher than that in the third quartile because participants in Q5 used dental services that required a large number of visits per episode, such as orthodontic and prosthodontic treatments.

### 3.3. GEE Analysis

Table 2 shows the results of the GEE analyses. Model 1 was targeted at patients who traveled by vehicle, and Model 2 was targeted at patients who traveled by both vehicles and on foot. Model 3 included the geographical variables in Model 2. Travelling to the dental institution on foot might be limited to within the range of the person’s living area. Since there is no difference from the point of view of the consumer in the movement within the living area, considering the use of dental care by walking as a patient’s preference may not be appropriate. Therefore, it was excluded from the GEE analysis. Models 1 and 2 showed similar trends, indicating that the travel time for non-NHI-covered services took approximately 2 to 3 times longer than for NHI-covered service visits. Model 2 confirmed that travel time for non-NHI-covered services was approximately 3.4 times as long as it took to receive implant treatment, 3 times as long as gold inlay and resin filling, and 2.3 times as long as orthodontic treatment compared to NHI-covered services. Patients residing in rural counties traveled longer than large-city residents (Model 3), and income showed a statistically significant association with travel time, but the coefficient was close to zero, indicating that its influence on travel time was almost negligible.

### 3.4. Travel Time According to Income Quintile by Dental Care Service

**Figure 2:**
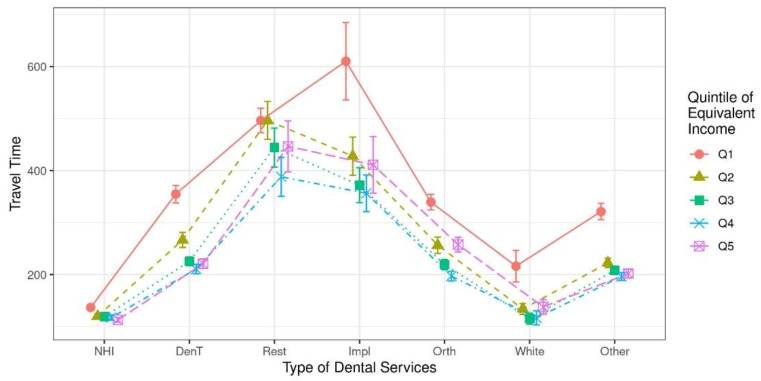
Travel time according to income quintile by dental care service.

The GEE results for equivalence income did not appear to have a meaningful effect on travel time, contrary to what was expected, based on previous studies. The present study showed travel time according to income quintile by seven dental service groups (Figure 2). The first quintile (Q1) traveled longer to receive all types of dental service. In particular, the travel time for the implant in Q1 was the longest. The dental care utilization of Q1 was clearly different from that of other quintiles, but there was no statistically significant difference between the remaining quintiles. The confidence intervals of Q1 did not overlap in any type of dental service, except gold inlay and resin filling with others. 

## 4. Discussion

Travel time, which is usually proportional to travel distance [46], is a cost for patients, especially when insurance coverage for healthcare services is comprehensive or the proportion of out-of-pocket expenses is not large [47]. Travel time also indicates the patient’s preference when selecting treatment [13]. In this study, the travel time for non-NHI-covered services increased compared to that for NHI-covered services. For example, implant and gold inlay and resin filling took approximately three times longer than NHI-covered treatment. These findings suggest that NHI-covered treatment was often provided near the place of residence, but non-NHI-covered services, which usually incurred high costs per episode, were served at the expense of opportunistic travel time. 

Orthodontic services took approximately 2.3 times longer to travel to compared to NHI-covered treatment. Orthodontic services improve appearance, promote oral health, and enhance self-esteem [48]. Moshkelgosha et al. [49] argued that the desire to receive high-cost and high-quality orthodontic treatment might differ depending on the socioeconomic background. Recommendations from friends or relatives and referral to a specific orthodontist were reported to serve as an option to select orthodontic treatment. Local residence and increased travel distances were not barriers to accessibility for orthodontic treatment in Medicaid-enrolled children and adolescents [11]. In this study, the increased travel time for orthodontic treatment was similar to the results of previous studies. Patients with high educational attainment preferred to travel for longer times to find orthodontists who were likely to provide high-quality services.

Dental implants have been an alternative dental service for prosthodontic treatments since the early 2000s, and the dental implant services as NHI benefits in 2014 have expanded the patient base in terms of treatment outcome satisfaction [50]. Dental insurance reform, which expanded the number of targets, provided NHI-covered implant services for up to two teeth by covering 30% of out-of-pocket expenditure [51]. Out-of-pocket expenditure for implant treatment was not as high as for other alternatives with higher satisfaction. Dental implants were evaluated as a cost-effective service from the patient’s point of view. The ease of information retrieval using online searches for dental institutions and implant services has itself contributed to the increase in dental implants [52]. In these circumstances, the number of patients receiving implant treatment has rapidly increased. The decisions made by patients after recognizing them could be interpreted as preferences [52].

Education showed an inverse relationship with travel time. Probst et al. [53] reported that the higher the education level, the shorter the travel time. Farlina and Maharani [54] found that low education levels were positively correlated with frequency of dental care utilization. In this study, different models showed different results, as shown in Table 2. Information acquisition and the utilization of dental services might not be directly proportional to educational background. Lee and Chang [55] reported that approximately 60% of university students had higher oral health literacy than junior high school graduates, but 40% had a lower oral health literacy. Our findings are similar to those reported by Lee and Chang [55]; however, for the highly educated group, the opportunity cost due to long visit times might be considered as important [56].

Jean et al. [57], who studied the travel time for dental care in socioeconomically disadvantaged groups living in remote communities, and Cao et al. [58], who reported the travel time for access to preventive dental care, showed that the lower the group, the greater the travel time. Seo et al. [59] reported that the NHI dental policy benefited the out-of-pocket spending of low-income households and the elderly. Table 2 of this study provides evidence to support this point. When dividing the income into five quintiles in Figure 2, no significant difference in travel time is shown by dental treatment among the other quintiles except for the lowest income quintile, and the lowest group in the quintile travels the longest distance regardless of the type of service. From these data, two interpretations were possible for the high-income groups. First, if it took a long time to travel for dental services, high-income groups might prefer not to travel far because the opportunistic cost was large in proportion. Second, many dental offices already provided preferred dental services near the patients’ homes, so they did not need to travel far. In fact, as of 2015, 75% of registered dentists practiced in metropolitan areas. By contrast, the frequency of dental care utilization was higher in the first quintile than in the fifth quintile. The frequent visits and long travel times in the first income quantile were seen as a result of poor oral health conditions and the search for affordable treatments by patients [13,53,60,61].

As a proxy for the distribution of dental resources in this study, rural county residents had a greater travel time for dental services than those living in large city districts, indicating geographical differences. Previous studies reported that the uneven distribution of dental offices became an obstacle to accessibility to dental care services [53,62,63]. When comparing Model 2 without regional variables and Model 3, there was a small difference in the coefficients. The role of regional variables in the effect of travel time on dental service selection was not large, meaning that the influence of uneven dental resources on dental care utilization was minor. It could be interpreted that within the same geographical locations, an increase in travel time was due to patient preference, and that the influence of the covariates was not significantly affected within the same region.

When selecting a dental clinic, patients often consider the opinions of family members or acquaintances, or an Internet search. Depending on limited information, they choose a dental institution that meets their dental needs. According to previous research that reported the use of mobile applications (apps) in relation to the accessibility of dental institutions in Korea, most apps are commercial and made for business [64]. Internet-based information produced by public institutions, such as ‘Finding Local Clinic,’ is very limited at the time of writing. A barrier of digital literacy may exist in the use of mobile apps. However, if a mobile app that provides crucial dental treatment information along with the location of the nearest dental office using location-based services is considered [65], it will have a positive effect on access to dental care. 

The main limitation of this study was that travel time might not reflect actual variations due to recall bias. However, since travel time was a response to multiple visits by patients, it was not only judged through the subjective memory of one visit but could rather be close to the objective travel time. Additionally, since the KHP was not a kind of claims data for the National Health Insurance Service, when information about the service type was collected, it depended on the respondents. Depending on the dental knowledge of the respondents, service types might not coincide with dental treatments. However, since the study utilized episodes rather than individual visits, the dental services used in this study would likely reflect actual treatment details.

## 5. Conclusions

In this study, the effect of travel time as a means of measuring the patient’s preference was examined. Travel times differed, depending on the type of dental service and the patient’s socioeconomic status. This preference was prominent in implant and orthodontic treatment and appeared as a result of reflecting the patient’s rational choice and health literacy in terms of dental care. The study’s findings suggest that if the dental patients required NHI-covered treatment, they preferred shorter travel times and were less prudent in choosing a healthcare provider. However, if the patients needed elective dental services, much more distant dental institutions (or providers) were considered. As one of the aspects of dental management, dental providers can deepen their relationship with patients through information as to their competitiveness and service quality improvement. This can have a positive impact on patients’ autonomous choice of health care institution as one of the factors that can overcome the accessibility barriers to health care use such as travel distance.

## Figures and Tables

**Table 1:** Travel time of transportation mode, based on the type of dental treatment and patient characteristics.

Items	Categories	Frequency (*n* = 53,963)	Travel Time by Walking *n* = 25,895 (48.0)	Travel Time by Vehicle *n* = 28,068 (52.0)	
		No. (%)	Median		*p*-Value ^3^
Type of dental service	NHI-covered ^1^	18,774 (34.8)	30	60	<0.001
	Denture and Fixed Bridge	11,075 (20.5)	60	120	
	Gold Inlay and Resin Filling	1792 (3.3)	180	330	
	Implant	1590 (2.9)	130	270	
	Orthodontic	9098 (16.9)	60	120	
	Tooth Whitening	2342 (4.3)	40	80	
	Others	9292 (17.2)	80	140	
Gender	Male	24,279 (45.0)	40	90	<0.001
	Female	29,684 (55.0)	50	120	
Age group	<20	14,244 (26.4)	36	60	
	20–65	27,724 (51.4)	60	120	
	>65	11,995 (22.2)	80	180	
Marital status	Married	24,641(45.7)	40	80	<0.001
	Single	29,322 (54.3)	60	120	
Educational attainment	≤Elementary	19,363 (35.9)	40	90	<0.001
	Middle/High	21,180 (39.2)	60	120	
	≥College	13,420 (24.9)	40	100	
Job	No	24,698 (45.8)	60	140	<0.001
	Yes	29,265 (54.2)	40	80	
Private dental insurance	No	53,763 (99.6)	42	100	0.498
	Yes	200 (0.4)	60	90	
Medical aid recipients	No	53,369 (98.9)	40	100	<0.001
	Yes	594 (1.1)	60	190	
Equivalence income ^2^	Q1 (first quantile)—lowest	9546 (17.7)	60	160	<0.001
	Q2 (second quantile)	10,676 (19.8)	50	120	
	Q3 (third quantile)	11,018 (20.4)	40	90	
	Q4 (fourth quantile)	11,460 (21.2)	40	80	
	Q5 (fifth quantile)—highest	11,263 (20.9)	40	90	
Geographical region (*n* = 46,933)	Large city district	27,505 (58.6)	42	100	<0.001
	Rural counties	5202 (11.1)	60	135	
	Small and medium-sized cities	14,226 (30.3)	40	90	

^1^ NHI-covered included dental episodes for NHI-covered dental services, such as conservative, periodontal, endodontic, extraction, and sealant care; ^2^ income unit (1000 KRW); ^3^ *p*-values were calculated by median test.

**Table 2:** Results of GEE analysis, including variables associated with travel time.

Items	Categories	Model 1 (Travel Time by Vehicle) *n* = 10,524 (Person) 28,068 (Episode)		Model 2 (Total Travel Time) *n* = 16,256 (Person) 53,963 (Episode)		Model 3 (Total Travel Time) *n* = 13,367 (Person) 46,933 (Episode)	
		Exp (Coef)	*p-*Value	Exp (Coef)	*p-*Value	Exp (Coef)	*p-*Value
Type of dental services (Ref. NHI-covered)	Denture and Fixed Bridge	2.08	0.000	2.19	0.000	2.24	0.000
	Gold Inlay and Resin Filling	2.65	0.000	2.97	0.000	3.00	0.000
	Implant	3.22	0.000	3.37	0.000	3.37	0.000
	Orthodontic	2.23	0.000	2.27	0.000	2.26	0.000
	Tooth Whitening	1.69	0.000	1.73	0.000	1.77	0.000
	Others	1.93	0.000	2.06	0.000	2.09	0.000
Gender (Ref. Male)	Female	1.07	0.045	1.04	0.169	1.05	0.117
Age group (Ref. 20–65)	<20	0.57	0.000	0.53	0.000	0.55	0.000
	>65	1.28	0.000	1.33	0.000	1.30	0.000
Marital status (Ref. Married)	Single	0.87	0.001	0.90	0.004	0.90	0.004
Educational attainment (Ref. ≤ Elementary)	Middle/High	1.27	0.000	1.16	0.000	1.22	0.000
	≥College	1.15	0.022	1.06	0.202	1.13	0.029
Job (Ref. No.)	Yes	0.84	0.000	0.87	0.000	0.87	0.000
Private dental insurance (Ref. No)	Yes	0.97	0.868	1.18	0.273	1.12	0.515
Medical aid recipients (Ref. No)	Yes	1.49	0.001	1.45	0.002	1.50	0.004
Equivalence income		1.00	0.002	1.00	0.003	1.00	0.007
Geographical region (Ref. Large city districts)	Rural counties					1.38	0.000
	Small and medium-sized cities					1.02	0.428

NHI-covered included dental episodes for NHI-covered dental services, such as conservative, periodontal, endodontic, extraction, and sealant care; Ref. refers to the reference group for categorical variables; Exp (coef) refers to the exponential of the GEE coefficient; unit of equivalent income was 100,000 KRW.

## Data Availability

Restrictions apply to the availability of these data. Data were obtained from the Korea Institute for Health and Social Affairs and are available at https://www.khp.re.kr:444/eng/main.do (accessed on 10 February 2022) with the permission of the Korea Institute for Health and Social Affairs.
